# In-Ovo Imaging with Ostrich Eggs: Eggshell Attenuation in CT and Limitations of Organ Dosimetry

**DOI:** 10.1007/s11307-025-02065-6

**Published:** 2025-11-18

**Authors:** Christian Kühnel, Tabea Nikola Schmidt, Olga Perkas, Marta Pomraenke, Julia Greiser, Martin Freesmeyer, Thomas Winkens

**Affiliations:** 1 Clinic for Nuclear Medicine, University Hospital Jena, Am Klinikum 1, 07747 Jena, Germany; 2 Working Group for Translational Nuclear Medicine and Radiopharmacy, Clinic of Nuclear Medicine, University Hospital Jena, Am Klinikum 1, 07747 Jena, Germany

**Keywords:** Computed tomography, Ostrich eggshells, Dose estimation, In-ovo imaging

## Abstract

Ostrich eggs have recently attracted interest as an alternative model in preclinical nuclear medicine imaging. The ability to be used in clinical PET/CT (positron emission tomography/computed tomography) systems and their ethical profile are advantageous over conventional rodent models and other avian systems. Nevertheless, concerns regarding radiation exposure during repeated CT (computed tomography) imaging of developing embryos remain inadequately addressed. This study aimed to characterize the attenuation impact of eggshells in ostrich eggs and to evaluate the potential for organ-specific dose assessment. A representative ostrich egg was selected from a cohort of 168 eggs and used to construct a dimensionally matched 3D-printed phantom. Organ weights of 83 embryos were documented on development day (DD) 37 to provide a basis for future organ-level dosimetric modeling. Thermoluminescence dosimeters (TLDs) were positioned along the z-axis within both the egg and phantom, and CT dose distributions were measured using a clinical PET/CT system. The mean absorbed dose in the real egg was 16.3 ± 2.0% lower than in the phantom, attributable to radiation attenuation by the 1.89 ± 0.12 mm thick eggshell. CTDI (computed tomography dose index) values remained stable across developmental stages (DD 0–37). Our findings confirm that the ostrich eggshell exerts a significant shielding effect during CT imaging. While ostrich eggs are suitable for serial in-ovo imaging, embryo positioning remains a major limitation for precise dosimetry. Organ weight data enable potential use of AI (artificial intelligence)-based modeling to improve spatial dosimetry accuracy. This study provides essential groundwork for dose optimization and radioprotection in preclinical imaging protocols using ostrich eggs.

**Supplementary Information:**

The online version contains supplementary material available at 10.1007/s11307-025-02065-6.

## Background

The utilization of ostrich eggs for in-ovo imaging has witnessed a marked increase in the context of preclinical nuclear medicine research. Areas of application include the characterization of in vivo distribution of newly developed radiopharmaceuticals on PET/CT and the monitoring of embryonal development using CT [1, 2]. Ostrich eggs have emerged as a promising model, offering several advantages over both traditional animal models like rodents and established animal replacement models like chicken eggs [2, 3]. The utilization of the knowledge and experience derived from the well-established chicken model could facilitate the establishment and further development of ostrich eggs in the context of clinical research [4, 5]. Due to their size, which is comparable to the cranium of a human infant, ostrich eggs allow for imaging on standard clinical scanners, including clinical PET/CT, thus avoiding the need for specialized and cost-intensive small-animal devices [2]. Moreover, fewer ethical constraints are associated with their use, since the practice of research on eggs prior to hatching is not classified as an instance of animal experimentation under the majority of national and international guidelines. [6].

This new field of preclinical imaging rose specific concerns regarding radiation exposure of ostrich embryos and its influence on physiologic embryonal development, especially in the case of serial imaging studies at different development stages [1]. It is unclear, whether repeated CT examinations pose a relevant radiation exposure to ostrich embryos, thus potentially influencing later stage experiments.

The purpose of this brief article is to provide a comprehensive overview of the salient aspects of dosimetry in ostrich eggs, with a particular emphasis on the impact of eggshell radiation absorption including dosimetric measurements on egg-shaped phantoms. Furthermore, we identify limitations in organ dosimetry regarding preclinical imaging using ostrich eggs.

## Methods

### Eggs

First, data of 168 ostrich eggs were analyzed in order to identify a suitable reference egg which served as ex-vivo model. To identify a representative egg for phantom-based dosimetry, we selected the specimen whose combination of shell thickness and weight was closest to the cohort medians. Intra-ovo circumferential variability of the shell thickness was not directly measured in this study. Based on prior reports and visual inspection, we assumed this variability to be negligible relative to the observed inter-individual variation [1]. Ostrich eggs were obtained from two local ostrich farms, located 20 km and 53 km from the research facility, respectively. Artificial incubation was conducted using an automatic tilting incubator with four hatches (Sofie3, J. Hemel Brutgeräte GmbH, Verl, Germany) with constant incubation properties at 36.5 °C and 25% humidity as described before [7]. All eggs (fertilized and unfertilized) were incubated for 37 d and ostrich embryos were euthanized after a final experiment as part of a larger research project. Egg weight was determined using a precision balance (Kern-FCB12Ki, KERN&SOHN GmbH, Balingen-Frommern, Germany). The thickness of the eggshell was measured using a 2 × 2 cm sample cut from the egg tip using a rotating cutter (Dremel, Bosch Powertools B.V., Breda, Netherlands) and a calibrated micrometer with a ball attachment for non-planar surfaces (Micrometer IP54 digital, KSM-PREISSER, Gammertingen, Germany), a well-established method for assessing eggshell thickness [8]. The aforementioned handling steps were part of a larger research project regarding in-ovo imaging using ostrich eggs. This also included serial CT scans on different development days (DD), i.e. DD 0, 10, 19, 22, 25, 28, 31, 34, and 37.

### Phantom

In analogy to the reference egg, dimensions, weight and eggshell thickness of 168 ostrich embryos were used to create a digital phantom model using CAD software (Inventor Professional 2022, Autodesk Inc, San Rafael, California, USA). The phantom geometry was derived from CT-based segmentation of the reference egg and subsequently transferred into a CAD and stereolithography format. The phantom was printed with a mid-range 3D printer (Ultimaker2 +, Ultimaker, Geldermalsen, Netherlands) from polylactic acid (PLA) with a HU of 138 ± 12 [9] a biopolymer widely used in medicine and research modelling [10], due to its tissue-equivalent attenuation properties [11]. PLA allows a fast and precise fabrication process and provides a watertight container for experimental filling. Different studies demonstrated that PLA exhibits attenuation properties comparable to water or soft tissue in the diagnostic energy range [12, 13]. In contrast, the calcified eggshell is characterized by a much higher effective atomic number and density, leading to stronger attenuation. Consequently, the PLA does not replicate the absorption characteristics of the eggshell, but rather provides a conservative model that underestimates the dose reduction which would occur inside an egg.

A 0.4 mm large extruder was selected for fabrication of the printed phantom parts with a minimal layer thickness of 0.1 mm and accuracy of 0.0125 mm. The printed compounds were subjected to a post-fabrication measurement process including the usage of a digital caliper gauge (profi scale precise PS 7215, accuracy 0.01 mm, Burg-Wächter, Wetter, Germany) and CT scans (focal spot size 0.9 × 1.1 mm/7°, isotropic resolution using z-Ultra-High-Resolution technology 30 lp/cm and 0.17 mm). This ensured minimal discrepancy in dimensions between design and reference egg and guaranteed the reliability of the conducted dose comparisons.

### CT Acquisition

All measurements were performed on a PET/CT system (Biograph mCT, Siemens Healthineers, Erlangen, Germany) equipped with a 40-slice diagnostic CT (Definition AS from Siemens Healthineers, source-detector distance: 1085.6 mm) with 120 keV and 200 mAs. To ensure consistent image quality and accurate dose measurements, a calibration of the CT system was performed prior to imaging. Two sets of scan parameters were used for calibration. With a tube current of 350 mA, a collimation of 12 × 1.2 mm, and a rotation time of 1.0 s, the computed tomography dose index (CTDI) was 21.98 mGy/100 mAs in air and 15.18 mGy/100 mAs in a head phantom. A second calibration profile, using a higher tube current of 420 mA, a finer collimation of 20 × 0.6 mm, and a shorter rotation time of 0.5 s, resulted in a CTDI of 22.86 mGy/100 mAs in air; no corresponding value was available for the head phantom under these conditions. The underlying CTDI_Head_ value (for objects ~ 16 cm in diameter) was 0.0152 cGy/mAs, comparable to values reported for small animal devices (0.03 and 3.7 cGy/mAs) [14]. The reference egg and the phantom were both filled with water to simulate soft tissue attenuation. An air cell was created on the top of the inside by incomplete filling. For reproducibility, the eggs and the phantom were consistently placed upright with the air bubble upwards in a custom holder (PU 145, Polyurethane 145 kg/m^3^, Schurg GmbH, Bad Wildungen, Germany) with a Hounsfield unit of −845 HU. This air cell orientation is consistent with that employed in several studies [1, 15].

### TLD Dosimetry

A total of nine dosimeters (TLD XD-700; Li:Mg,Ti; 100 mg/cm^2^, Thermo Fischer Scientific Inc. Waltham, Massachusetts, USA), with detector dimensions of 3.175 × 3.175 mm and 0.381 mm thickness were utilized for dosimetry along the z-axis of the egg and phantom. The detection and recognition limits were 0.03 mSv and 0.02 mSv, respectively. Read-out and analysis were performed externally by the “Landesanstalt für Personendosimetrie und Strahlenschutzausbildung” (LPS, Köpenicker Straße 325, Berlin) with an automated TLD reader (HARSHAW 6600 CCD). Calibration was performed according to the LPS protocol with traceability to national standards. Reproducibility of the TLD system is σ < 5% for doses above 100 µSv. Dose assessment was based on the personal dose equivalent H_p_(0.07), as only differences between the reference egg and phantom were considered relevant, without applying correction factors. Dosimeters were placed on a rack along the z-axis inside of the egg, in order to detect the CTDI deviation depending on the related overlying eggshell thickness.

### Statistical Analysis

All statistical analyses were performed using the R statistical computing environment (version 4.3.1) within the RStudio interface (Posit Software, Boston, MA, USA). Continuous variables are reported as mean ± standard deviation (SD), unless stated otherwise. Normality of data distributions was assessed using the Shapiro–Wilk test. A two-sided paired t-test was applied to compare dose measurements obtained from the reference egg and the 3D-printed phantom, based on the assumption of normal distribution and paired placement of TLDs along the z-axis. The significance level was set at α = 0.05. Data visualization was conducted using the base graphics system and the ggplot2 package (version 3.4.0) in R. Graphical representations were optimized for clarity and comparability of dose profiles between measurement conditions. No corrections for multiple testing were applied, as only a single primary hypothesis was tested.

## Results

### Egg and Phantom

**Fig. 1:**
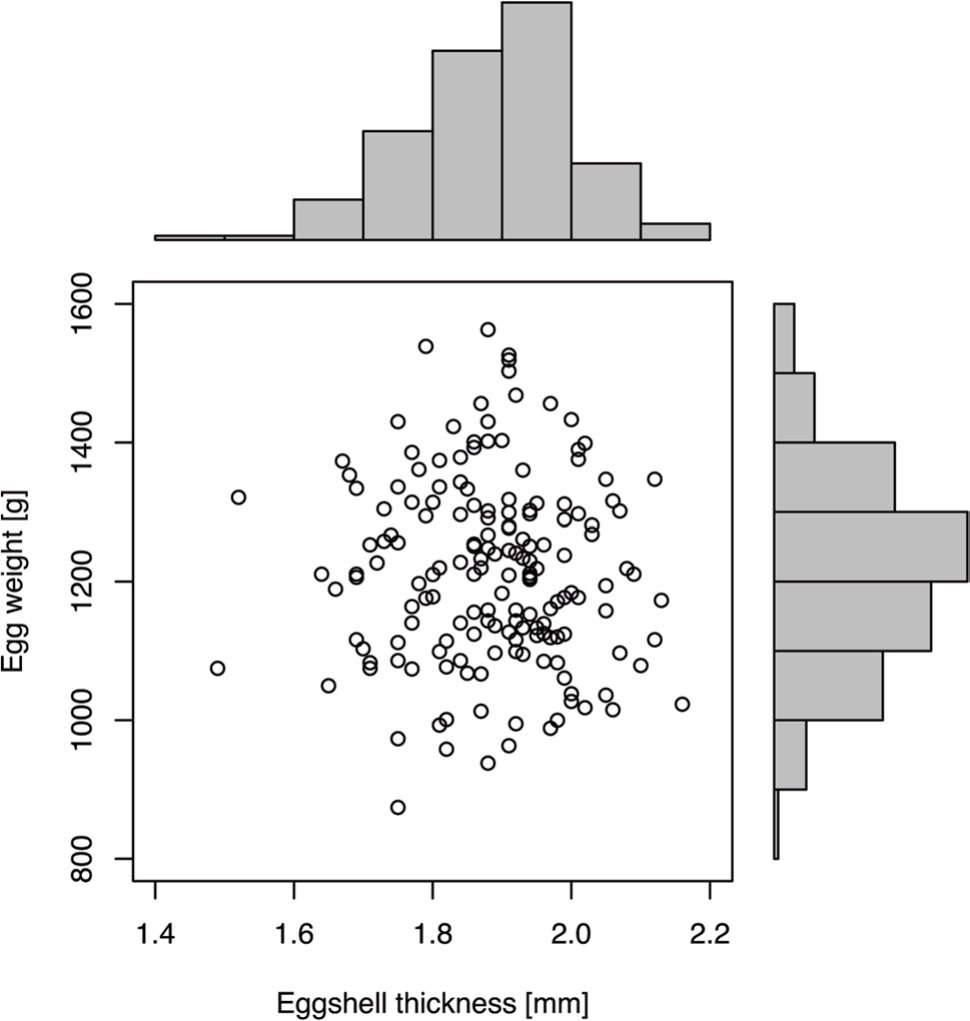
Distribution of eggshell thickness and corresponding egg weight for 186 ostrich eggs

83 fertilized and 85 unfertilized ostrich eggs did not show significant differences regarding CTDI_vol_ (33.61 ± 1.16 mGy; median: 33.88 mGy; range: 30.89—35.88 mGy), neither between different eggs nor between different DD. The mean weight on DD 37 was 1215.1 ± 133.8 g (1211 g; 874—1563 g) with an interquartile range (IQR) of 1118—1303 g and the eggshell thickness showed a mean of 1.89 ± 0.12 mm (1.90 mm; 1.49—2.16 mm) with an IQR 1.81—1.96 mm (Fig. 1). Weight data showed an average weight loss of 0.3% per day for both, fertilized and unfertilized eggs, showing no significant difference.

**Fig. 2:**
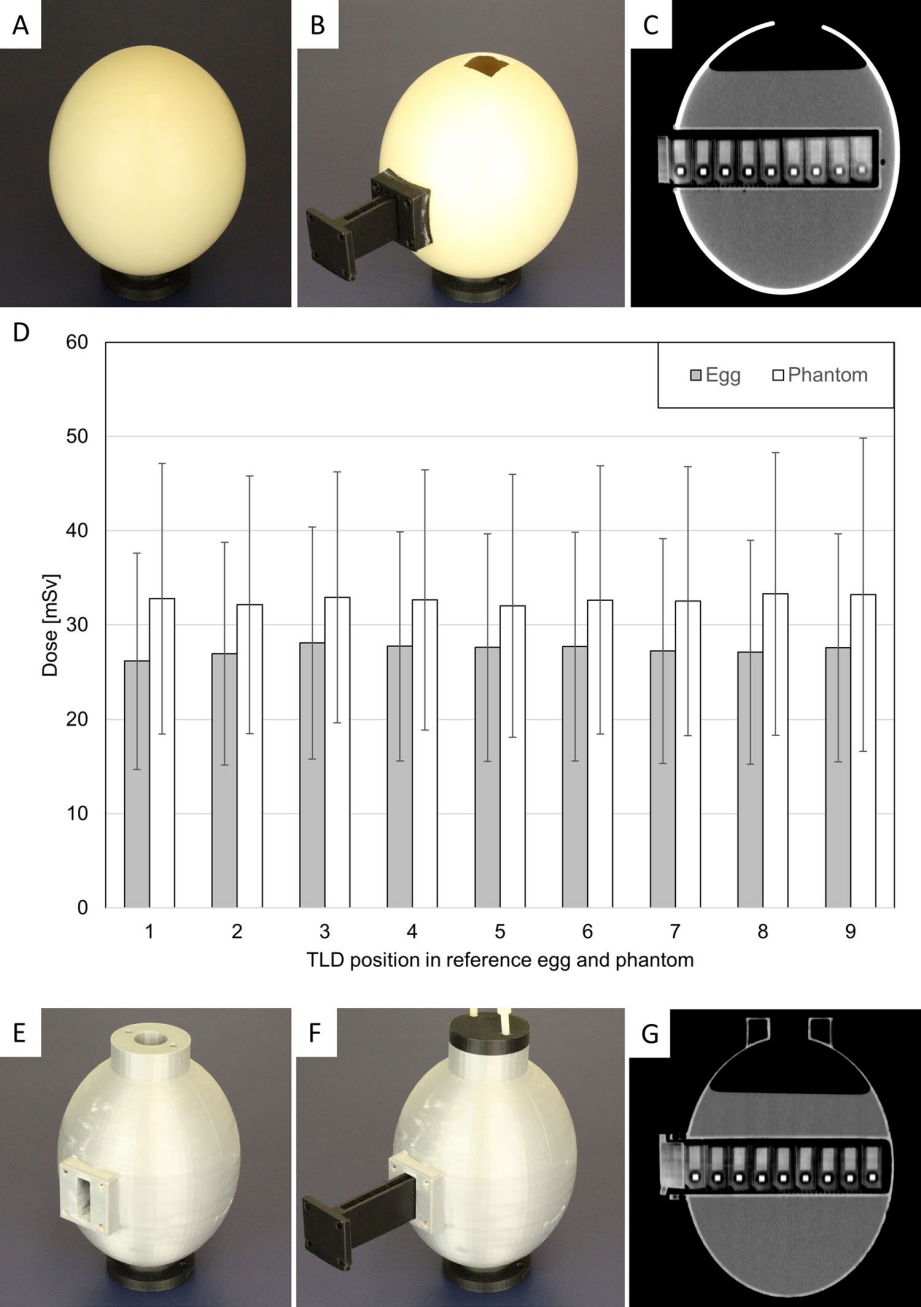
Reference egg with a weight of 1216 g (**A**). Once the top window had been removed and the egg emptied, a printed PLA insert was attached to the egg (**B**), ensuring that the TLDs were reproducibly positioned within the TLD holder for each measurement in the CT (**C**). Dose comparison between egg and phantom was shown (**D**). The egg phantom (**E**) also has a lid and a PLA insert to keep the TLDs dry when filled with water (**F**). The reproducible measurements obtained from the CT were comparable to those of the real egg (**G**). Selected CT parameters for egg and phantom (**C, G**) were 120 kV, flat filter type, pitch factor 0.8, increment 0.3 mm, slice-thickness of 0.6 mm, 200 mAs, nominal collimation width single 0.6 mm and total 6 mm, rotation time 1 s

The printed compounds of the phantom exhibited conformity with the constructed dimensions, falling within the prescribed tolerance range. Figure 2A-C shows the reference egg before preparation (A), after insertion of the TLD rack (B) and on a CT image (C). The phantom consisted of three main elements: an egg replica with an internal shaft (Fig. 2E) to hold the TLD-rack, and a lid on top to prevent leakage (Fig. 2F). The positioning of the TLDs in the phantom was analogous to the positioning in the reference egg (Fig. 2C, G).

### TLD Measurements

For the reference egg and the phantom, three sets of ten proof-of-principle measurements were performed with nine TLDs positioned along the z-axis, respectively (Fig. 2C, G). The average dose for one CT-scan per TLD of the reference egg was 27.4 ± 0.6 mSv (27.6 mSv; 26.2—28.1 mSv), while the average dose for the phantom was 32.7 ± 0.4 mSv (32.7 mSv; 32.0—33.3 mSv) (Fig. 2D). A paired t-test demonstrated a statistically significant difference corresponding to a mean attenuation of 16.3 ± 2.0% (16.2; 13.8—20.2) due to the eggshell (p < 0.001), which is attributable to the eggshell thickness of the reference egg. Details are shown in the supplemental material.

### CTDI Values

Longitudinal analyses of CTDI values from DD 0–37 reveal no variation over time, suggesting consistent radiation dose across the developmental stages of the egg (data not shown). This stability ensures the reliability of CT imaging in longitudinal studies by minimizing dose-dependent artifacts. The uniform CTDI supports the suitability of the imaging protocol for comparative embryological research. As the object thickness (eggshell) remains unchanged during embryo development, the CTDI also does not change over time.

### Organ Weights

In order to collect data for future organ-specific dosimetry, the mean measured organ weights of 83 ostrich embryos were documented after euthanatization on DD 37, with a particular focus on their correlation with the ostrich's body mass. Details are shown in Table 1 and thus provide a basis for discussion of the influence of the eggshell on organ radiation and could contribute to further investigations regarding radiation exposure of specific body areas.

**Table 1:** Organ masses of ostrich embryo removed from fertilized eggs on DD 37 and relation to respective bodyweight of ostrich embryo in grams. Bone mass was determined via threshold-based CT segmentation

	**Collected values**	
**Organ**	**Mass, mean ± SD [g]**	**rel [g/gBW]**
Brain	3.72 ± 0.67	0.7
Heart	1.97 ± 0.25	0.4
Stomach	8.48 ± 2.56	1.6
Small intestine	4.74 ± 1.24	0.9
Large intestine	6.48 ± 2.20	1.2
Kidneys	2.98 ± 0.69	0.6
Liver	5.87 ± 1.16	1.1
Skeleton	199.31 ± 16.97	38.1
Thyroid	0.21 ± 0.02	< 0.1
Others (residuary organs, tissue)	289.24 ± 37.24	55.3
Embryo	523 ± 35.60	100
Egg weight without embryo	692 ± 111.80	N/A

## Discussion

The use of chicken, and more recently ostrich eggs, as substitutes for rodent models in preclinical imaging has emerged as a promising alternative. In-ovo imaging not only serves as an animal replacement model but also reduces regulatory burden [1]. CT scanners for small animals are equipped with a flat panel detector. In contrast, human scanners are equipped with spiral CT, a configuration that reduces the dose and time required for adequate imaging [16]. The large gantry bores of these scanners can accommodate ostrich eggs, which renders them particularly advantageous in preclinical imaging, specifically the field of development of new radiopharmaceuticals.

In our setup, the 3D-printed phantom filled with water served as the baseline reference without a shell, since PLA and water approximate soft-tissue attenuation but lack the calcified shell. The observed 15–20% higher dose in the phantom therefore directly reflects the additional attenuation of the eggshell. This discrepancy underscores the dosimetric significance of the shell and substantiates the phantom’s role as a reproducible, standardized comparator, independent of biological variability.

**Fig. 3:**
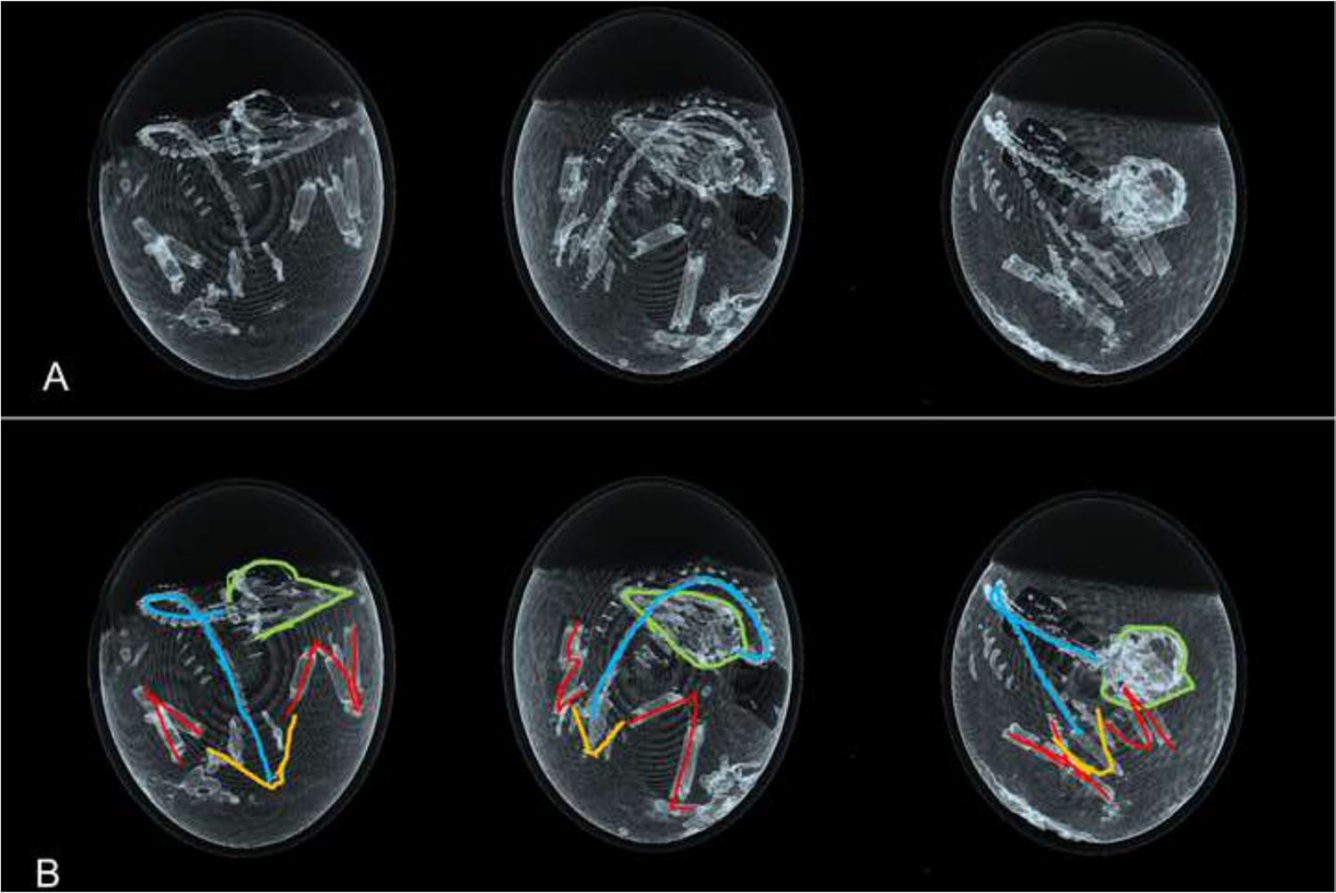
Highly variable position of three different ostrich embryos on DD 37 (**A**). **B** shows color-coded position of head (green), spine (blue), pelvis (orange) and lower extremity (red)

Despite its advantages, imaging using ostrich eggs presents challenges. While motion artifacts during biokinetic measurements can be controlled by means of narcotization, the non-reproducible interindividual position of the embryo inside the eggshell remains a major limitation for in vivo dosimetry and kinetic modelling [4, 7, 17] (Fig. 3). The embryo accounts for up to 40% of the egg’s weight and, due to transport and rotation during incubation, its position cannot be consistently replicated. In contrast, positioning in mice or humans is straightforward and verifiable. Beyond the qualitative visualization, embryo positioning could in principle be quantified using basic spatial metrics. These include the normalized elliptical distance of the embryo centroid from the egg center, the angular deviation of the embryo’s major axis relative to the longitudinal egg axis, or sector-wise frequencies of embryo localization. While these descriptors permit preliminary numerical characterization of spatial variability, further systematic investigations are required before such parameters can be robustly implemented as input for voxel-based Monte Carlo simulations or related modelling approaches.

Concerns regarding eggshell thickness variability between different eggs—and its impact on measurement reliability—are unfounded, as Falk et al. (2018) demonstrated that thickness changes over time are negligible (8).

This study demonstrates, in a proof-of-principle experiment with one representative egg/phantom pair, that the eggshell measurably attenuates radiation dose. While repeated TLD measurements ensured precision for this specific specimen, the reliance on a single reference egg inherently limits the generalizability of the findings. The representativeness of this egg was defined by its proximity to the cohort medians of shell thickness and weight; however, it cannot capture the full biological variability within the population. Future work should therefore include multiple biological replicates to account for natural variation in shell thickness and composition and to confirm the observed attenuation effects across the broader cohort. Nevertheless, the present data provide a robust experimental baseline that can be used as a foundation for dosimetric estimations and simulation studies.

We present data which serves as prerequisite for organ dosimetry. Another approach analogous to the Altman phantom could also be considered, involving freezing the egg, slicing it to insert TLDs into organs, and reassembling it for layer-specific dose assessment. However, practical issues such as material loss during slicing and heat generation complicate this approach. This study provides the first organ mass data derived from dissections of fertilized eggs. While organ mass alone is insufficient for organ dosimetry, voxel-based Monte Carlo simulations or AI-based embryo repositioning, these data represent a valuable baseline which, in combination with imaging and segmentation, may support future modelling approaches.

## Conclusion

Ostrich eggs have the potential to serve as a viable substitute for conventional animal models in preclinical studies. The examination of eggshell thickness showed that this factor cannot be disregarded in dose considerations. However, the highly variable position of the ostrich embryo in the egg leads to large inaccuracies in the precise dosimetric estimation of anatomical and metabolic imaging. Further studies incorporating AI should investigate whether organ weights, as measured in this study, could enable ostrich eggs to facilitate more detailed clinical modelling.

## Supplementary Information

Below is the link to the electronic supplementary material.

Supplementary file1 (DOCX 781 KB)

## Data Availability

The datasets used and analyzed during the current study are available from the corresponding author on reasonable request.

## Abbreviations

AI: Artificial Intelligence
cGy: Centigray
CT: Computed Tomography
CTDI: Computed Tomography Dose Index
DD: Development Day
HU: Hounsfield Unit
Li:Mg, Ti: Lithium Fluoride (Isotope)
LPS: Landesanstalt für Personendosimetrie und Strahlenschutzausbildung
mA(s): Milliampere(seconds)
mGy: Milligray
mSv: Millisievert
PET/CT: Positron Emission Tomography / Computed Tomography
PLA: Polylactic acid
TLD: Thermoluminescence dosimeter

## References

[1] Winkens T, Christl A, Kuehnel C, Ndum F, Seifert P, Greiser J et al (2021) In-ovo imaging using ostrich eggs—evaluation of physiological embryonal development on computed tomography. Acta Zool 103(4):492–502

[2] Freesmeyer M, Kuehnel C, Opfermann T, Niksch T, Wiegand S, Stolz R et al (2018) The use of ostrich eggs for in ovo research: making preclinical imaging research affordable and available. J Nucl Med 59(12):1901–1906 29934406 10.2967/jnumed.118.210310

[3] Makanya AN, Jimoh SA, Maina JN (2023) Methods of in ovo and ex ovo ostrich embryo culture with observations on the development and maturation of the chorioallantoic membrane. Microsc Microanal 29(4):1523–1530 37488818 10.1093/micmic/ozad060

[4] Heidrich A, Wurbach L, Opfermann T, Saluz HP (2011) Motion-artifact-free in vivo imaging utilizing narcotized avian embryos in ovo. Mol Imaging Biol 13(2):208–214 20552287 10.1007/s11307-010-0355-4

[5] Wurbach L, Heidrich A, Opfermann T, Gebhardt P, Saluz HP (2012) Insights into bone metabolism of avian embryos in ovo via 3D and 4D ^18^F-fluoride positron emission tomography. Mol Imaging Biol 14(6):688–698 22422564 10.1007/s11307-012-0550-6

[6] (2010) Directive 2010/63/EU of the European Parliament and of the Council of 22 September 2010 on the protection of animals used for scientific purposes. Off J Eur Union 20(276):33–79

[7] Freesmeyer M, Hermeyer H, Kuehnel C, Perkas O, Greiser J, Witte OW et al (2022) In-ovo imaging using ostrich eggs: biomagnetism for detection of cardiac signals and embryonal motion. Exp Biol Med (Maywood) 247(12):996–1004 35466741 10.1177/15353702221082046 PMC9265528

[8] Falk K, Møller S, Rigét FF, Sørensen PB, Vorkamp K (2018) Raptors are still affected by environmental pollutants: Greenlandic peregrines will not have normal eggshell thickness until 2034. Ornis Hung 26(2):171–176

[9] Van der Walt M, Crabtree T, Albantow C (2019) PLA as a suitable 3D printing thermoplastic for use in external beam radiotherapy. Australas Phys Eng Sci Med 42(4):1165–1176 31728939 10.1007/s13246-019-00818-6

[10] DeStefano V, Khan S, Tabada A (2020) Applications of PLA in modern medicine. Eng Regenerat 1:76–87 10.1016/j.engreg.2020.08.002 PMC7474829 38620328

[11] Kuhnel C, Seifert P, Mulik C, Winkens T, Freesmeyer M (2020) 3D printing of fillable individual thyroid replicas based on nuclear medicine DICOM data used as phantoms for gamma probe calibration. Nuklearmedizin 59(1):12–19 31856284 10.1055/a-1070-9874

[12] Ma X, Buschmann M, Unger E, Homolka P (2021) Classification of X-ray attenuation properties of additive manufacturing and 3D printing materials using computed tomography from 70 to 140 kVp. Front Bioeng Biotechnol 9:763960 34912790 10.3389/fbioe.2021.763960 PMC8666890

[13] Villani D, Rodrigues O, Campos LL (2020) Dosimetric characterization of 3D printed phantoms at different infill percentages for diagnostic X-ray energy range. Radiat Phys Chem. 10.1016/j.radphyschem.2020.108728

[14] Carlson SK, Classic KL, Bender CE, Russell SJ (2007) Small animal absorbed radiation dose from serial micro-computed tomography imaging. Mol Imaging Biol 9(2):78–82 17285239 10.1007/s11307-007-0080-9

[15] Perkas O, Pomraenke M, Porwoll V, Kuhnel C, Wiegand S, Herrmann KH et al (2025) In ovo model with emu eggs as novel alternative to animal testing in preclinical imaging research. EJNMMI Res 15(1):118 40960561 10.1186/s13550-025-01314-7 PMC12443669

[16] Boone JM, Velazquez O, Cherry SR (2004) Small-animal X-ray dose from micro-CT. Mol Imaging 3(3):149–158 15530250 10.1162/15353500200404118

[17] Perkas O, Schmidt A, Kuehnel C, Greiser J, Hermeyer H, Klingner C et al (2024) Different narcotic gases and concentrations for immobilization of ostrich embryos for in-ovo imaging. Exp Biol Med (Maywood) 249:10037 38854792 10.3389/ebm.2024.10037 PMC11157058

