# Supplementary material for: In-Ovo Imaging with Ostrich Eggs: Eggshell Attenuation in CT and Limitations of Organ Dosimetry
Source: Mol Imaging Biol. 2025 Nov 18;27(6):863–9. doi: 10.1007/s11307-025-02065-6 (PMC12804239; doi:10.1007/s11307-025-02065-6)
Supplement: Supplementary file 1 — Supplementary file1 (DOCX 781 KB) [file 11307_2025_2065_MOESM1_ESM.docx]

**Supplementary material**


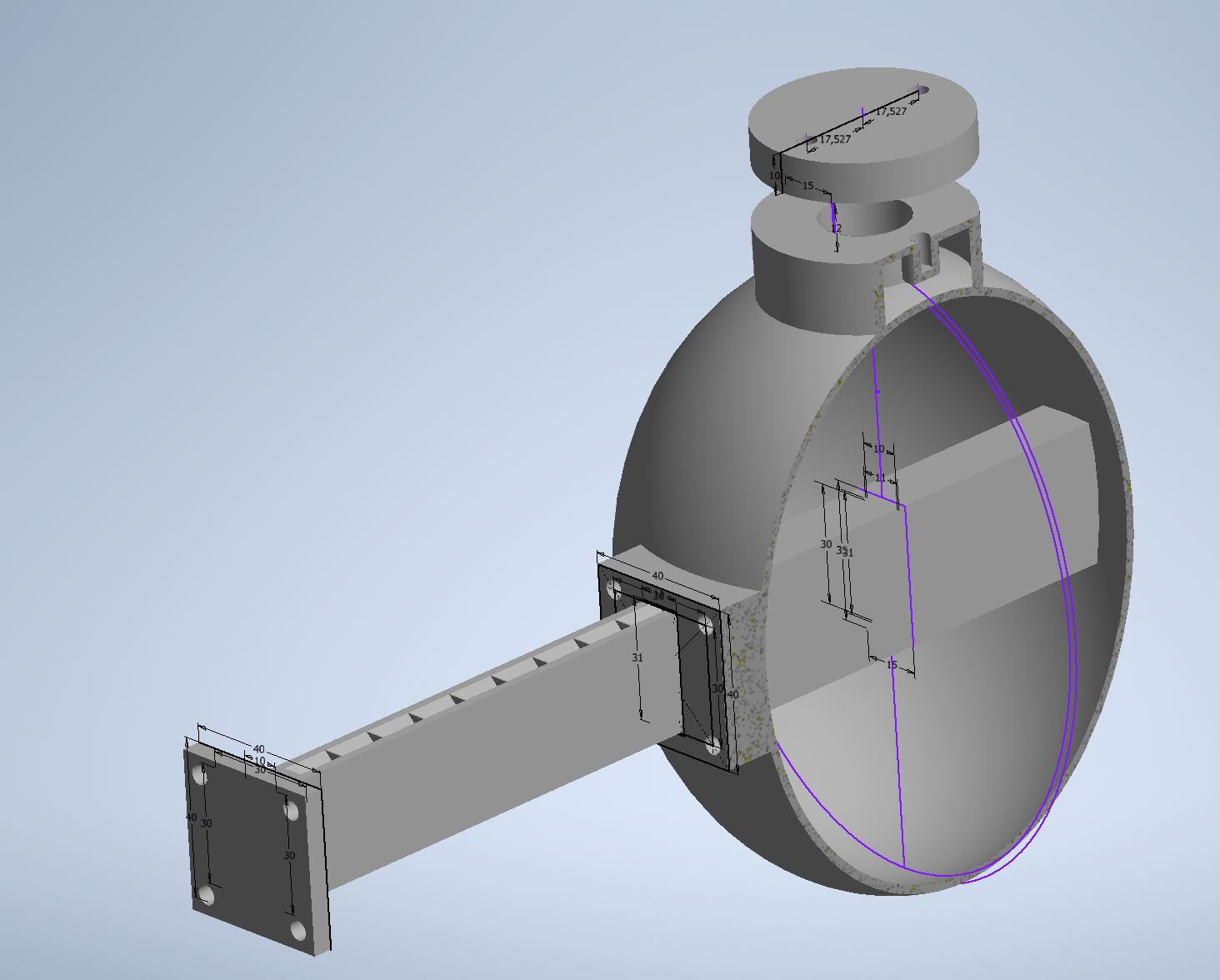


**Figure**: The phantom was reconstructed using the CAD software Autodesk Inventor based on physiological reference dimensions. The spherical main compartment represents the egg fillable with water, while the rectangular insert was modelled to hold the thermoluminescent dosimeters (TLDs), allowing reproducible placement along predefined positions.
